# Precision medicine technology hype or reality? The example of computer-guided dosing

**DOI:** 10.12688/f1000research.20489.1

**Published:** 2019-10-01

**Authors:** Thomas M. Polasek, Sepehr Shakib, Amin Rostami-Hodjegan

**Affiliations:** 1 Certara, 100 Overlook Center, Suite 101, Princeton, NJ, 08540, USA; 2 Centre for Medicines Use and Safety, Monash University, Melbourne, Victoria, Australia; 3 Department of Clinical Pharmacology, Royal Adelaide Hospital, Adelaide, South Australia, Australia; 4 Discipline of Pharmacology, Adelaide Medical School, University of Adelaide, Adelaide, Australia; 5 Centre for Applied Pharmacokinetic Research, University of Manchester, Oxford Road, Manchester, M13 9PL, UK

**Keywords:** Precision medicine, precision dosing, computer-guided dosing, model-informed precision dosing, personalized medicine, individualized drug therapy

## Abstract

Novel technologies labelled as ‘precision medicine’ are targeting all aspects of clinical care. Whilst some technological advances are undeniably exciting, many doctors at the frontline of healthcare view precision medicine as being out of reach for their patients. Computer-guided dosing is a precision medicine technology that predicts drug concentrations and drug responses based on individual patient characteristics. In this opinion piece, the example of computer-guided dosing is used to illustrate eight features of a precision medicine technology less likely to be hyperbole and more likely to improve patient care. Positive features in this regard include: (1) fitting the definition of ‘precision medicine’; (2) addressing a major clinical problem that negatively impacts patient care; (3) a track record of high-quality medical science published via peer-reviewed literature; (4) well-defined clinical cases for application; (5) quality evidence of benefits measured by various clinical, patient and health economic endpoints; (6) strong economic drivers; (7) user friendliness, including easy integration into clinical workflow, and (8) recognition of importance by patients and their endorsement for broader clinical use. Barriers raised by critics of the approach are given to balance the view. The value of computer-guided dosing will be decided ultimately by the extent to which it can improve cost-effective patient care.

## Introduction

Precision medicine is defined as ‘treatments targeted to the needs of individual patients on the basis of genetic, biomarker, phenotypic, or psychosocial characteristics that distinguish a given patient from other patients with similar clinical presentations’ ^1^. Novel technologies labelled as precision medicine are targeting all aspects of clinical care with the promise of better healthcare for all via better treatment of the individual. Examples are diverse, but include companion molecular diagnostics for pharmaco- and immuno-therapy in oncology and hematology ^2^, pharmacogenomic-guided drug and dose selection ^3^, and artificial intelligence to stratify clinical risk and treatment options (IBM Watson Health). Whilst some technological advances are undeniably exciting, widespread clinical application beyond specialized centers is limited, particularly outside the United States. No doubt, the words ‘precision medicine’ create business opportunities, advance academic careers, have political appeal, and resonate with the media and public. However, many doctors at the frontline of healthcare view precision medicine as merely indulgent fine-tuning for a privileged few rather than a ‘game-changer’ for all. In this opinion piece, the example of computer-guided dosing, sometimes called clinical pharmacometrics and/or model-informed precision dosing (MIPD) ^4, 5^, is used to illustrate eight features of a precision medicine technology less likely to be hyperbole and more likely to improve patient care. Barriers raised by critics of the approach are also given to balance the view.

### (1) Defined as precision medicine

First, the novel technology should fit the definition of precision medicine quoted above ^1^, rather than just applying common sense more specifically to an individual patient e.g., fitness devices linked to applications that track and encourage exercise. Computer-guided dosing predicts drug concentrations (‘exposure’) in the body based on individual patient characteristics such as age, weight and gender. Some models also incorporate physiological and molecular characteristics, including drug metabolizing enzyme and transporter activities and how these change in disease states or in the presence of interacting drugs. More sophisticated models predict drug responses based on dose-exposure-response relationships, although such modelling is relatively less advanced compared with exposure and requires further understanding of many pathophysiological states e.g., disease progression models etc. The dose required for each patient to achieve the target exposure is then relatively straightforward to determine, with the ultimate goal of accurately predicting drug responses. In cases where the pathophysiology is relatively simple or well understood (e.g., bacterial cell killing and antiviral effects), prediction of drug responses is relatively advanced ^6^. Critics of the approach use examples where the pathophysiology is complex or poorly understood (e.g., neurology and psychiatry), which makes accurate modelling of drug responses difficult. Models can give dose predictions prior to starting drug treatment, but they are particularly powerful for dose adjustment via Bayesian feedback after initial drug exposure and/or a biomarker of response is known in a particular patient ^7^.

### (2) Addresses a clinical problem

The novel technology should target a well-defined clinical problem that negatively impacts patient care. Many patients receive no benefits from drug treatment. Worse still, drugs cause patient harm, costing about US $42 billion per year globally ^8^. One of the key reasons for these problems is that drug exposure may vary more than 10-fold for the same drug at the same dose in different patients. Computer-guided dosing adjusts for between-patient variability in drug exposure. The prescribing focus changes from selecting a dose, to selecting a dose needed to achieve a target exposure, which is one-step closer to response ^9^. This goes against the industry culture of ‘one-dose-fits-all’, which is adopted for commercial reasons. Some prescribers may also underplay the role of dose as a cause of patient harm. It is accepted that dose is just one of many factors that contribute to adverse drug effect susceptibility, including **I**mmunological, **G**enetic, demographic ( **A**ge and **S**ex), **P**hysiological, **E**xogenous factors (e.g., drug-drug interactions) and **D**isease and disorders (e.g., renal failure), giving the mnemonic **I GASPED** ^10^. But amongst these factors, dose is the major one that can, and therefore should, be modified. Thus, the clinical problem of patient harm from drugs is more likely to be reduced, rather than ‘solved’ by computer-guided dosing.

### (3) Track record of high-quality medical science

The novel technology should not, on closer review, be so novel. Computer-guided dosing was first proposed in 1969 for anticoagulation ^11^, with seminal publications demonstrating clinical utility in the 1970s for digoxin ^12^. Since the millennium, affordable ‘-omics’ technologies (genomics, proteomic and metabolomics), superior analysis of biological samples, improved medical imaging, and powerful computers to analyze data, have enabled many sources of between patient variability in drug exposure and/or response to be identified, understood and then modelled e.g., warfarin. A clear narrative in the peer-reviewed literature adds confidence that a precision medicine technology is not just a ‘flash-in-the-pan’. However, a down-side of peer-reviewed literature is an over-emphasis of success stories, and the field of computer-guided dosing is probably also influenced by this publication bias.

### (4) Clinical use is well-defined

The novel technology should have a well-defined clinical application. A classic sign of precision medicine hype is the ‘oversell’, usually from a commercial provider, which occurs when *potential* clinical utilities are promoted beyond the scope of *actual* clinical utilities. Computer-guided dosing is yet to be subject to such promotion, although several private companies have entered the market in recent years. Indeed, many doctors are unaware of computer-guided dosing or the clinical cases for which the approach is helpful. It is important to note that the patient, disease and drug characteristics that combine for high impact computer-guided dosing are well-defined ^4^. The approach is best for difficult to dose drugs in difficult to dose patients when the clinical stakes are high. In other words, the use of narrow therapeutic index drugs when safer options are unavailable/unacceptable in pregnant women, neonates, children, those with severe organ dysfunction, the hemodynamically unstable, the frail elderly, and patients with multiple co-morbidities on polypharmacy. These patient groups are typically excluded from clinical trials that establish the recommended dose(s), so doctors are ‘flying blind’ with dosing when they must use drug treatment. For precision medicine technologies, knowing which patients, which diseases, and which drugs not to study is equally as important as knowing which clinical cases to study. This allows efficient resource allocation and the evidence of clinical utility to grow more rapidly. An important criticism of precision medicine is that it is not a ‘game-changer’ for all, however, all significant changes in clinical practice have begun in a narrow and clearly defined group of patients.

### (5) Evidence of benefits

The novel technology should be supported by several independent studies that include a range of clinical, patient-reported and health economic endpoints. For computer-guided dosing, there is work on clinical outcomes with antibiotics in the critically ill, with immunosuppressants and chemotherapy in serious pediatric illnesses, and with chemotherapy in adult oncology. For example, recent randomized controlled studies comparing computer-guided dosing of paclitaxel versus body surface area-based dosing in patients with non-small cell lung cancer show trends for decreased toxicities (e.g., grade 4 hematological toxicities and neutropenia and > grade 2 neuropathy) without compromising efficacy ^13, 14^. More high quality studies of this nature are required to generate clinical evidence supportive of computer-guided dosing more broadly ^15^. Data are especially needed for commonly used narrow therapeutic index drugs, including those started by medical specialists and continued by general practitioners, such as the direct oral anticoagulants and psychotropics (clozapine, lithium).

### (6) Strong economic drivers

The novel technology should catch the eye of business developers and healthcare administrators. In the last two decades, computer-guided dosing has revolutionized drug development because it saves the pharmaceutical industry time and money ^7, 16^. The appeal of translating this success to healthcare is the economic driver for the private sector. Unsustainable public spending on healthcare is shifting the re-imbursement of drugs from a supply-based model to one that rewards positive clinical outcomes. This is forcing a re-think on the ‘one-dose-fits-all’ strategy that has previously served drug developers well. Publicly funded incentives would then be in place to find the best drug at the best dose for a particular patient. Unfortunately, strong economic drivers also create an environment for ‘sharks in the water’, further emphasizing the need for high quality evidence of benefits (section 5).

### (7) User friendliness

The novel technology should be embraced by doctors. Disruption to clinical workflow should be minimal and considered ‘worth the effort’ i.e., the sweet spot between clinical speed and clinical accuracy is retained. Decision support tools (DSTs) for computer-guided dosing have been integrated successfully into various in-house and commercially available electronic health records. An example with high doctor satisfaction is a DST built for busulfan, a narrow therapeutic antineoplastic drug used to prepare pediatric patients for bone marrow transplantation ^17^. However, such examples are rare, and a major barrier to computer-guided dosing will be familiarity and acceptance of DSTs by everyday prescribers who remain unconvinced of the clinical need. A ‘forcing function’ for adoption may be the automatic inclusion of such tools within e-prescribing modules of commercially available electronic health records.

### (8) Patients drive broader clinical uptake

Finally, the novel technology should be embraced by patients and their advocates. Broader and faster uptake of technology occurs when ‘consumers’ experience benefits and spread the word. Indeed, some precision medicine developers are now by-passing the medical profession entirely with direct-to-consumer strategies e.g., pharmacogenomic testing. There are no published data thus far on the appeal of computer-guided dosing to patients, so future studies should include endpoints to capture their perspectives ^5^.

## Conclusion

Many doctors resent the insinuation of precision medicine that their clinical practice is not individualized enough and therefore not good enough. The disparity between innovative clinical applications and the reality of resource-constrained clinical practice is considered too wide. Features of precision medicine technologies more likely to bridge this disparity are illustrated here using the example of computer-guided dosing. This approach is now 50 years old and has matured to the point where clinical implementation is expected beyond specialist units in academic medical centers. The value of disruptive technologies in clinical medicine, including computer-guided dosing, will be decided ultimately by the extent to which they can improve cost-effective patient care.
