# Supplementary material for: Precision medicine technology hype or reality? The example of computer-guided dosing
Source: F1000Res. 2019 Oct 1;8:1709. [Version 1] doi: 10.12688/f1000research.20489.1 (PMC6852323; doi:10.12688/f1000research.20489.1)
Supplement: Peer review history

# Peer review: Reviewer response for version 1

**DOI:** 10.5256/f1000research.22521.r55228

**Published:** 2019-11-11

**Authors:** Richard N. Upton

**Affiliations:** 1 School of Pharmacy and Medical Sciences, University of South Australia, Adelaide, SA, Australia

This seems a sensible opinion article discussing the requirements for the successful implementation of precision medicine using drug dosing as an example. 

 Two minor comments.

1. Under Introduction heading (1),  bacterial  cell  killing  and  antiviral  effects are not examples of pathophysiologies. These are examples of mechanisms of action
2. Under Introduction heading (6), "Unsustainable public spending on healthcare ": Why public here? Private spending via insurance can also be unsustainable.

I confirm that I have read this submission and believe that I have an appropriate level of expertise to confirm that it is of an acceptable scientific standard.

# Peer review: Reviewer response for version 1

**DOI:** 10.5256/f1000research.22521.r54521

**Published:** 2019-10-30

**Authors:** Daniel F. B. Wright

**Affiliations:** 1 School of Pharmacy, University of Otago, Dunedin, New Zealand

This commentary provides a well-argued and timely overview of important features of precision medicine, specifically focused on computer guided dosing. This is an important contribution at a time when model-informed precision-dosing is being touted (once again, or ‘finally’ depending on your perspective) as a means of improving patient outcomes and mitigating drug related harm. This argument is not new, but the well-considered points raised by the authors suggest that the conversation around this topic is ready to be escalated. Indeed, there is a general sense in the literature – whether real or not – that proponents of this approach are making headway with broad clinical implementation in some settings.

 It is important to emphasize that computer-guided dosing – as used throughout the paper - is a very broad term. Outputs of clinical pharmacometric analyses or model-informed precision dosing are not synonymous with computer based dosing, as implied by the authors in the introduction, but are simply examples. Almost anything can be put on a computer as part of decision-support system, including simple empirical dosing guidelines that may have been developed historically from less than robust analyses. The authors address this point somewhat under “(1) Defined as precision medicine” but I wonder if a clear statement indicating that the paper is referring to dosing guidance that has been derived from (hopefully) robust modelling analysis is warranted. These could be simple model-based dosing tables implemented in e-prescribing systems or purpose-built Bayesian feedback programmes. As the authors note, the models underpinning the outputs might be population pharmacokinetic models or mechanistic PBPK or systems pharmacology models (there are currently few examples of the latter).

 On a related note, the statement under “(2) addressed a clinical problem” that computer-guided dosing will adjust for between patient variability in drug exposure is not necessarily true. This is only the case of the dosing tool has been derived from a modelling analysis and/or is implemented in a Bayesian feedback system.

 The title seems to promise some debate about whether precision medicine is hype or reality. While the paper presents eight characteristics for precision medicine that are required for “reality”, i.e. to inform clinical care, there is only a passing reference to the hype. The title might need some consideration to ensure that it aligns with the content of the paper.

 A minor point. The eight features are nicely presented but (1) and (3) could be relabelled for clarity. “Defined as precision medicine” is a bit hard to understand on first read. Something like, for example, (1) “aligns to an accepted definition of precision medicine”, (3) “Derived from high-quality medicine science” are suggestions.

I confirm that I have read this submission and believe that I have an appropriate level of expertise to confirm that it is of an acceptable scientific standard.
